# Biological effects of COVID-19 on lung cancer: Can we drive our decisions

**DOI:** 10.3389/fonc.2022.1029830

**Published:** 2022-10-10

**Authors:** Beatrice Aramini, Valentina Masciale, Anna Valeria Samarelli, Roberto Tonelli, Stefania Cerri, Enrico Clini, Franco Stella, Massimo Dominici

**Affiliations:** ^1^ Division of Thoracic Surgery, Department of Experimental, Diagnostic and Specialty Medicine—DIMES of the Alma Mater Studiorum, University of Bologna, G.B. Morgagni—L. Pierantoni Hospital, Forlì, Italy; ^2^ Division of Oncology, Department of Medical and Surgical Sciences, University of Modena and Reggio Emilia, Modena, Italy; ^3^ Laboratory of Cell Therapy, Department of Medical and Surgical Sciences, University Hospital of Modena, University of Modena and Reggio Emilia, Modena, Italy; ^4^ Respiratory Disease Unit, Department of Medical and Surgical Sciences, University Hospital of Modena, University of Modena and Reggio Emilia, Modena, Italy

**Keywords:** COVID-19, pandemic, virus, lung cancer, genes, mutations

## Abstract

COVID-19 infection caused by SARS-CoV-2 is considered catastrophic because it affects multiple organs, particularly those of the respiratory tract. Although the consequences of this infection are not fully clear, it causes damage to the lungs, the cardiovascular and nervous systems, and other organs, subsequently inducing organ failure. In particular, the effects of SARS-CoV-2-induced inflammation on cancer cells and the tumor microenvironment need to be investigated. COVID-19 may alter the tumor microenvironment, promoting cancer cell proliferation and dormant cancer cell (DCC) reawakening. DCCs reawakened upon infection with SARS-CoV-2 can populate the premetastatic niche in the lungs and other organs, leading to tumor dissemination. DCC reawakening and consequent neutrophil and monocyte/macrophage activation with an uncontrolled cascade of pro-inflammatory cytokines are the most severe clinical effects of COVID-19. Moreover, neutrophil extracellular traps have been demonstrated to activate the dissemination of premetastatic cells into the lungs. Further studies are warranted to better define the roles of COVID-19 in inflammation as well as in tumor development and tumor cell metastasis; the results of these studies will aid in the development of further targeted therapies, both for cancer prevention and the treatment of patients with COVID-19.

## Introduction

The development and spread of COVID-19 in the last 2 years have compelled scientists to focus on devising strategies for treating the infected patients as well as for managing the resultant health care emergency (1, 2). This reduced the scientists’ ability to focus on other important diseases, such as cancer (3, 3). Hospitals and universities have had to remodel themselves in the last few years, modifying their work and shifting their focus to studying the SARS-CoV-2- and COVID-related pathways and features (4, 5). Given that patients with COVID-19 required urgent medication and hospitalization, those with other morbidities were deprioritized, and their surgeries were rescheduled. This has impacted the collection of biological samples (fluids and tissues) for research, especially in the field of thoracic oncology (6, 7). Moreover, some studies have shown that the incidence of lung cancer was higher among patients who contracted COVID-19 than among those who did not and that the risk of developing severe illness and death was greater among patients with lung cancer than among those with other cancers (8, 9). This has steered the scientific community toward studying the possible connections between SARS-CoV-2 and lung cancer (10). The increased incidence of lung cancer among patients with COVID-19 is likely attributable to the severe immunosuppression caused by the virus, changes related to the inflammatory components and the cascade of immunogenic events activated by the virus, and, finally but not less importantly, the possibility that the virus is oncogenic (11, 12). A particularly interesting concept is that that an external factor, such as a viral infection that causes multiple changes to the microenvironment (e.g., extracellular matrix remodeling and cell-to-cell interactions especially with the immune system), awakens dormant cancer cells from quiescence (13, 14). This state of dormancy is characterized by tumor cells that no longer undergo cell division but are inactive in the G0–G1 state; however, an appropriate stimulus can trigger proliferation (13, 14). This theory is related to drug resistance and metastatic niche activation, which should be taken seriously in the case of lung cancer, especially, due to its capacity to disseminate to primary organs (13, 14).

Hence, understanding the biology of infection with SARS-CoV-2 among patients with lung cancer and their response to COVID-19 vaccines is of utmost importance (15–17). One of the most intriguing aspects of COVID-19 is that the expelled respiratory droplets containing the virus may settle on different surfaces or be suspended in the air, thereby facilitating further contamination (18). Moreover, the virus can survive on several surfaces and in different organs and tissues, contributing to the contagiousness of the disease (19). Apart from the bronchopulmonary tree, SARS-CoV-2 RNA has been detected in the head, digestive tract, nervous system, heart, and blood vessels, among others (20). Therefore, considering the easy transmissibility and virulence of SARS-CoV-2, it is imperative to observe strict hand-washing hygiene (21).

Furthermore, the virus can quickly modify its nature, and scientists are thus using modern techniques, such as next-generation sequencing, for routine viral DNA analysis (22–24). This approach is used for liquid biopsy samples and in case of autopsies with suspected infections, following the biosafety level 3 guidelines (22–25). The use of liquid biopsies allows new insights, and regulations have been adopted to protect pathologists and technicians in the last few years (25, 26). However, because SARS-CoV-2 has a high degree of virulence, the use of pneumatic air tube transport systems to deliver pathology samples from the hospitals to the laboratories is not allowed (25, 26). This problem has been discussed in numerous countries, including the United States, Spain, Italy, Germany, Portugal, Denmark, Scotland, the Netherlands, and Slovenia, although some centers are still using pneumatics to transfer pathology samples (26). The challenges associated with establishing new procedures and rules for managing COVID-19 have helped introduce new approaches on a global scale, through online webinars and conferences, and adaptations that encompass different approaches to be followed at hospitals and in laboratories (27, 28).

Despite the numerous vaccines that have been administrated in countries worldwide, new variants continue to spread globally; moreover, although fewer devastating outcomes are being reported, the infection rate is still increasing, and the effects of this virus on the human body, particularly on the lungs, have not been fully elucidated (29). The scientists are still trying to understand the roles of immunity in the progression and resolution of COVID-19 as well as tumor development (30, 31). Several studies have been conducted recently to comprehend the possible roles of the virus in cancer growth; however, at the moment, the scientific community scientists have no definitive findings to share (10–12).

## SARS-CoV-2 damages the lungs

One of the most considered problems during the first wave of COVID-19 was the overcrowding in intensive care units (ICUs), which the governments decided to close for all non-COVID-19 patients (32). The primary symptoms of COVID-19 include acute respiratory failure and acute respiratory distress syndrome (ARDS), which are the main reasons for admission to the ICUs (33). Patients with such severe symptoms required protective low-tidal volume and mechanical ventilation, which is considered the standard of care for moderate-to-severe ARDS (33, 34). Moreover, anesthesiologists expressed the need to establish additional respiratory support strategies to ensure the optimal use of these machines at low volumes (35). The government discussed this problem with other countries, and several invasive and noninvasive devices were shipped from China, North America, and other countries (36).

In normal lung functioning, in which breathing is a cyclic process driven by respiratory muscles, viscoelastic tissues get deformed during normal inspiration and are relieved when the pressure returns to the initial situation during expiration (37). Under nonphysiological conditions, when the pulmonary tissue deformation is greater than normal, the resultant stress, represented by the transpulmonary pressure in association with the global strain, may affect the lungs (37). In fact, under pathological conditions, strain and stress may cause ventilator-induced lung injury (38). In particular, high volumes of nonphysiological conditions may be harmful to the lung, increasing the risk of mortality among patients with ARDS (38). Throughout the pandemic, anesthesiologists in ICUs and pneumologists have uncovered knowledge that has led to the identification of different approaches for caring for patients with COVID-19 (39).

The most important feature to consider in an injured lung is the inhomogeneity of ventilation, which increases tissue damage, resulting in severe worsening of pulmonary gas exchange, thus promoting respiratory insufficiency in patients with ARDS (40). This vicious circle increases the stress on tissues, intensifying lung damage. Moreover, the inhomogeneous distribution of the opening pressure may overstretch the lungs and cause aerated areas to connect with poorly aerated lung regions (38, 41). This phenomenon associated with COVID-19 infection caused severe problems in ventilation, resulting in several deaths during the last waves of the pandemic (42).

A new four-dimensional tomographic approach can analyze the biomechanical setting of the lungs, defining the volume distribution, especially in the nonventilated lung regions (43, 44). A recent study conducted using healthy rats with spontaneous breathing reported heterogeneity in the volume, with consequent quantification of the deformation of lung areas and progression in time (35). The different ventilation in the various lung regions may be attributable to strain and stress, although other factors, such as alveolar–capillary barrier integrity, were considered (37). Upon follow-up with micro-computed tomography images, comparing animals with spontaneous breathing through controlled and uncontrolled mechanical ventilation, moderate-to-severe lung injury was observed in the nonaerated lung compartments (37); furthermore, the study findings demonstrated significant progression of the regional volumetric strain and heterogeneity after spontaneous breathing, with subsequent damage to the lung alveoli (37). Other studies were conducted to analyze lung heterogeneity in association with the severity of ARDS and subsequent mortality, particularly in relation to alveolar wall disruption, hemorrhage, hyperemia, and inflammation (45–48). Recently, researchers attempted to define a gene expression pathway related to lung homogeneity, although the gene expression is generally related to specific lung regions, in contrast with the damage, which is ubiquitous (49). This can perhaps be explained by the fact that biomarkers are mainly water soluble and diffusible in blood and in bronchial secretions (50–52).

Gattinoni et al. defined two phenotypes characterizing patients with COVID-19 infection in the lung: “non-ARDS” or “type 1” and “ARDS” or “type 2” (53, 54). However, although mechanical support is very important for patients with COVID-19 infection, it is not feasible to advocate a single guideline for this condition (55). Rather, the approach needs to be tailored to every individual patient to prevent serious complications (55, 56).

The pathophysiology of this virus is often characterized by respiratory failure; it is variable with moderate-to-severe hypoxemia (57). Accordingly, as initial respiratory support, the primary treatment choices include oxygen therapy, high-flow nasal cannula, and noninvasive ventilation. If these methods fail, mechanical ventilation becomes mandatory for improving lung ventilation, which is compromised upon infection with SARS-CoV-2 (58). One of the main strategies that has been adopted in the ICU to promote oxygenation is that the patients are maintained in the prone position, which also improves the functional residual capacity and ventilation/perfusion. This approach is also used in awake patients, although the associated principles have not been fully defined (58, 59).

## Biological effects of SARS-CoV-2 on lung cancer

In the last 2 years, the biological differentiation of SARS-CoV-2 has resulted in several DNA mutations, leading to different levels of viral virulence, with variable clinical consequences for patients (60–62). Studies have reported increasing evidence of genetic susceptibility to SARS and to several genomic differences in COVID-19 patients, which facilitate the entrance of the virus in patients with cancer (63). Moreover, the immunity of patients with cancer is low, and they are at a higher risk of exposure to infections, including COVID-19 (64). The lungs are the primary target of novel coronaviruses, and the scientific community is trying to define the roles of genetic mutations and immunity in patients contracting COVID-19 and related cancers (65–67). However, because the associated processes are extremely complicated, it is currently not possible to determine whether this virus plays a role in cancer development, although COVID-19 combined with a weakened immunity creates favorable conditions for cancer development (68, 69).

**Figure 1:**
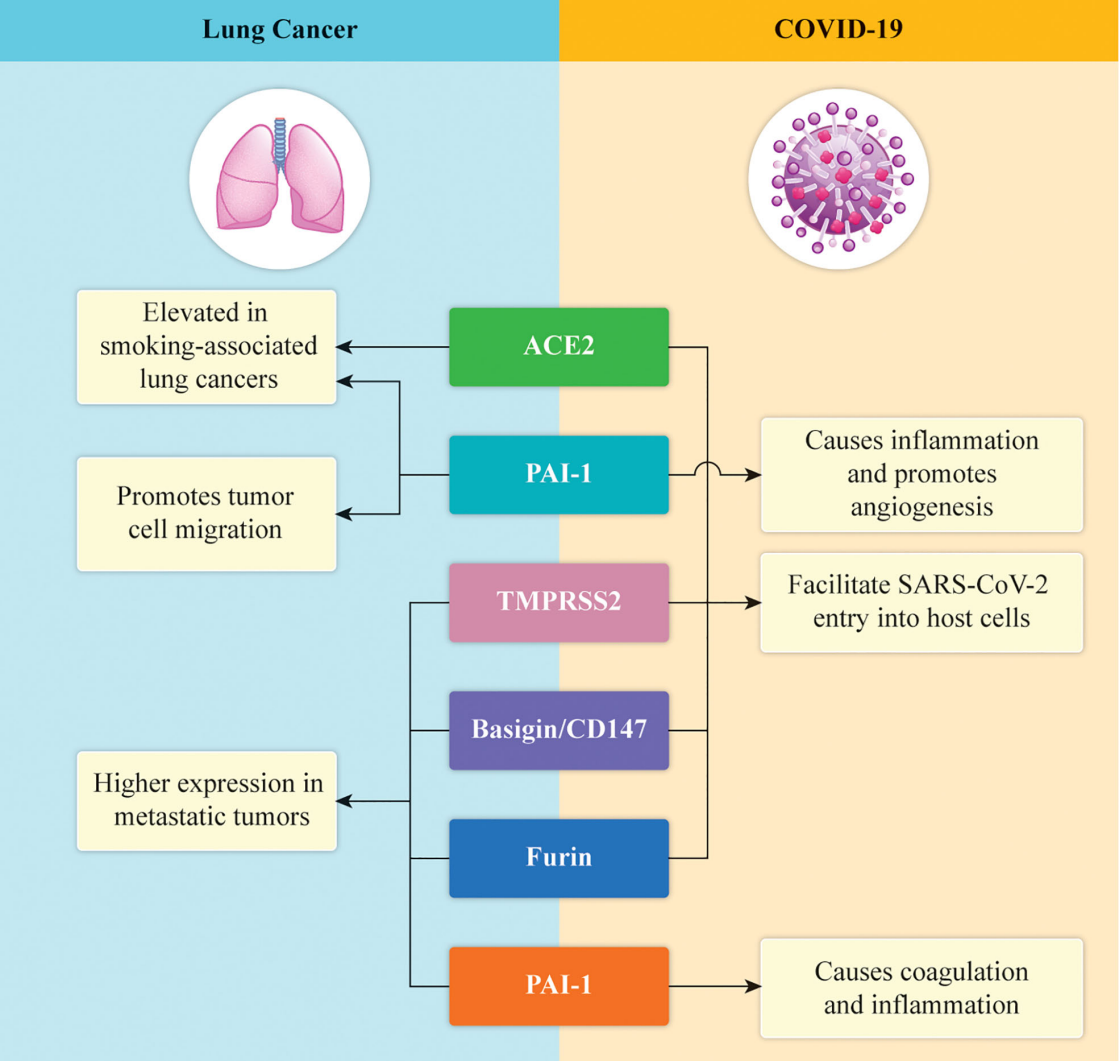
Graphical illustration of the expression of the key proteins in lung cancer and COVID-19. ACE2 and PAI-1 are expressed at high levels in lung cancer and cause inflammation and neo-angiogenesis in COVID-19. TMPRSS2 helps SARS-COV-2 to enter the host cells, whereas CD147 and FURIN are associated with genomic susceptibility to COVID‐19 in patients with lung cancer.

It has been recently reported that angiotensin-converting enzyme-2 (ACE2), and plasminogen-activator inhibitor type 1 (PAI-1), which have been proposed as receptors on mammalian host cells and seem to promote inflammation, angiogenesis, and coagulation, are the main molecular targets of the spike (S) protein of SARS-CoV-2 (70–72) (**Figure 1**). The serine protease transmembrane serine protease 2 (TMPRSS2), which seems to activate the S protein to enter into the cells, was also recently introduced as a molecular target (72–74). Moreover, the interaction between the virus and ACE2 receptor seems to be driven by the FURIN/PCSK3 cleavage of the S protein, facilitating viral invasion (75–77) (**Figure 1**). Basigin/CD147, a receptor glycoprotein of the immunoglobulin superfamily that acts as a mediator of the viral infection, was also reported recently (**Figure 1**) (78).

Besides studying these important protein receptors, which are considered to drive the entry of the virus into cells, researchers have also started to analyze the molecular profiles of the genes associated with the mutational patterns of the relevant targets (79, 80). These considerations were derived from the recent discovery of a possible correlation between the membrane proteins and lung adenocarcinoma (LUAD) and lung squamous carcinoma (LUSC) (81, 82). Specifically, these mutations have not only been detected but are also related to patient survival. In particular, a high percentage of genes targeting mutations have been found in the ACE2, TMPRSS2, CD147/BSG, and FURIN/PCSK3 genes (83).

For example, the TMPRSS2 gene expression was significantly reduced in patients with LUAD as compared with healthy patients, although the ACE2 expression was higher in the former group (84–86). Moreover, the TMPRSS2 gene expression was significantly reduced in patients with LUSC as compared with healthy patients, although the ACE2 expression was high in the patients with both LUAD and LUSC (87). No significant differences were noted in the expression of the CD147 and FURIN genes (88). Thus, defining the genomic susceptibility of patients with lung cancer to COVID‐19 remains challenging, and more experiments are warranted to assess the mechanisms of viral invasion, which may be the key to devising future treatments against the infections caused by SARS‐CoV‐2 (83, 89).

A significant amount of scientific literature has highlighted the possible involvement of ACE2 and TMPRSS2 gene mutations in cancer development (90). Specifically, in 2020, Stewart et al. (91), using different models of normal and malignant cells from the aerodigestive and respiratory tracts, respectively, found that ACE2 expression is highly correlated with the transcriptional, microRNA (miRNA), and metabolic signifiers of epithelial differentiation (91, 92). Moreover, the regulators of epithelial-to-mesenchymal transition (EMT) may play a role in the modulation of ACE2 expression. This biunivocal correlation may exert effects on infection caused by SARS-CoV-2 owing to the fact that it the virus reportedly increases EMT gene expression and metabolic alterations (93, 94). The association between COVID-19 and some gene variants directly affects the virus as well as lung involvement; moreover, the correlation between these proteins and the COVID-19-related EMT gene expression needs to be urgently explored by the scientific community to uncover the possible solutions and develop future targeted treatments (95).

## Immunomodulation of COVID-19 and lung cancer

One of the most important aspects related to COVID-19 is the cytokine storm, during which various inflammatory cytokines are developed, and the consequent cytokine release syndrome (CRS), which represents an acute attack of excessive cytokines from the immune system triggered by inflammatory responses and the immunity (96, 97). The CRS is characterized by high fever, erythema, edema, extreme fatigue, and nausea, which may also be associated with sepsis and multiple organ failure (98). The first approach by the cytokines and chemokines represents an important step against the viral infection; in particular, macrophages function as sentinels in the lungs, and SARS-CoV2 can infect these to induce this cascade as an early pathogenic mechanism (99, 100). The virus infects the dendritic cells, thereby inducing a cascade of cytokines and chemokines, including CCL3, CCL5, CCL2, and CXCL10 (23), which are the key components in the chemotactic approach induced by neutrophils, monocytes, and T cells, thereby calibrating a dysregulated response against infections caused by SARS-CoV-2 (101).

**Figure 2:**
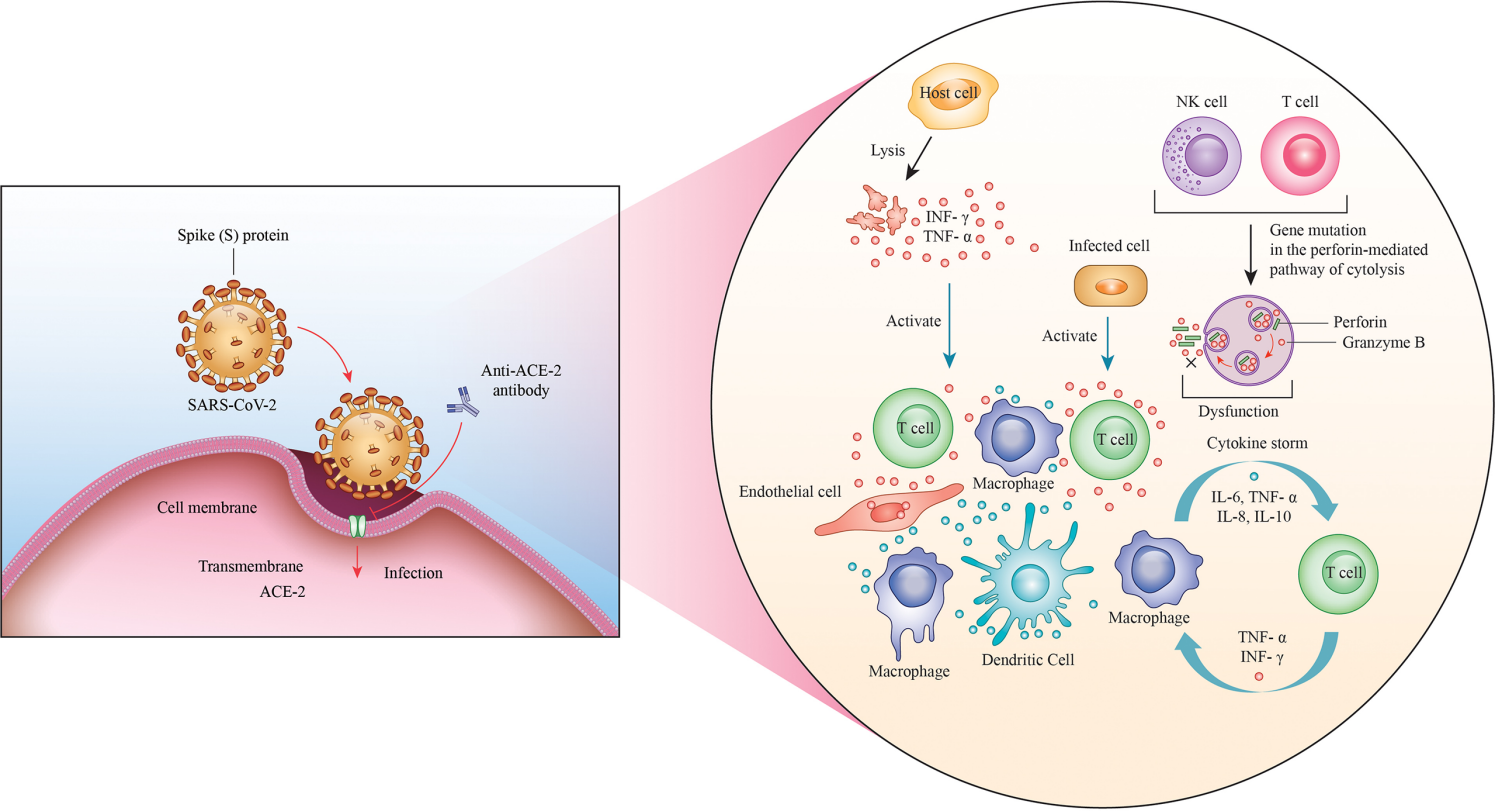
Invasion with SARS-CoV-2. The ACE2 receptor is the channel through which the virus attaches to the cells *via* its S protein and enters the cell. A massive immune response through the amplification of NK and T cells is thereby triggered, leading to the production of a wide range of cytokines, such as interferon-γ, tumor necrosis factor, interleukin (IL)-1, IL-6, and IL-18, which are responsible for the cytokine storm.

In particular, the immunodeficiency or the altered immune cell response induced by infection with this virus may lead to vivid outcomes among patients with cancer, owing to the complex relationship between the virus and cancer (102). Furthermore, patients with response have a higher risk of exposure and susceptibility to infections as compared with the normal population, making them more vulnerable to infection with SARS-CoV-2 (103). The main consequences of this factor include the overlapping of infections, an altered immune response, and faster progression of the virus and cancer; in addition to these aspects, a higher risk has been reported among patients with obesity, cardiovascular disease, hypertension, diabetes, and other comorbidities, which induce further complications during COVID-19 (104). Moreover, the scientific community has recently identified that the lung cancer population is at higher risk of contracting COVID-19 and that the aberrant expression of ACE2 in lung carcinoma renders the patients more susceptible to COVID-19 (105, 106). By definition, ACE2 is a regulator of the renin–angiotensin system and is located in a small subset of alveolar type II cells; its basic function is to convert angiotensin II into angiotensin (107, 108). Likewise, it has been reported that SARS-CoV-2 can attach to the ACE2 receptor through its S protein and thereby enter the cells (109, 110). Although ACE2 is also expressed in other organs such as the heart, kidneys, and intestine, the lungs are its target organ (111). Invasion by SARS-CoV-2 induces a lung immunoreaction against the virus, with the activation and amplification of host immunity, consequently inducing a cascade of cytokines, including interferon-γ, tumor necrosis factor, interleukin (IL)-1, IL-6, and IL-18, all of which play important roles in the toll-like receptor signaling pathway (**Figure 2**) (112, 113). This high level of cytokines induces ARDS and organ failure with consequent death of the patient; particularly, the presence of the virus increases lung cancer cell apoptosis, thereby downregulating the expression of ACE2 and causing altered vascular permeability, neutrophil infiltration, and lung edema (114). Interestingly, the maintenance of the ACE2 serum levels improves the survival of patients with lung cancer, increasing the immune system’s capacity to induct an inflammatory storm that can eliminate SARS-CoV-2 from the lungs (115). Finally, ACE2 may be considered a biomarker and future therapeutic target against COVID-19 infection, although several studies need to be conducted to confirm this aspect (116).

Apart from the importance of ACE2 in the pathogenesis of the novel coronavirus, a higher percentage of patients with lung cancer were found to have contracted COVID-19 (117). A recent study analyzed 2000 patients with COVID-19 and found that 1% of those had a history of lung cancer, indicating that patients with cancer may be susceptible to COVID-19 (118). A panel of genes, including SLC6A20, LZTFL1, CCR9, FYCO1, CXCR6, XCR1, ABO, RPL24, FOXP4, TMEM65, OAS1, KANSL1, TAC4, DPP9, RAVER1, PLEKHA4, and IFNAR2, were assessed for their mRNA expression to study a possible connection with COVID-19 and lung cancer (119). At the end of this analysis, immunohistochemistry combined with a comparison with the Human Protein Atlas helped validate only six genes (SLC6A20, FYCO1, FOXP4, TMEM65, XCR1, and OAS1) with significantly different protein expression levels (118). This finding suggests a potential genetic predisposition between COVID-19 and lung cancer, indicating that patients with lung cancer have a higher risk of contracting COVID-19 (120). Accordingly, a complex bioinformatics analysis assessing COVID-19 and different lung cancers was performed, also correlating with the severity of cancer, to apply knowledge and invest efforts to fight this pandemic infection, especially scouting out genetic association with it.

Because of this, an interesting aspect that needs to be considered is the use of anticancer drugs in patients with concomitant COVID-19 infection, contributing to the overall complexity of this scenario in the potential cross-interference between COVID-19 and lung cancer treatments (121). The scientific community is trying to define the main correlations among all of these aspects, although no definitive data are available currently (121, 122).

In this context, medical approaches, such as chemotherapy, immunotherapy, and radiotherapy, may play a dual role in affecting the targeted organs as well as the immunity of patients with cancer (123). In particular, immune checkpoint inhibitors (ICIs), which are considered promising against thoracic malignancies, may play important roles in inducing an immunomodulatory effect (124). The upregulation of T cells and the concomitant expression of PD-1, which can identify exhausted T cell subpopulations, both of which contribute to acute viral infection, have been reported in the early stage of COVID-19 infection. Accordingly, CTLA-4 or the PD-1/PD-ligand (L)-1 axis may enhance the expression of CD4 and CD8, reinforcing the exhausted T cells and better supporting the potential effect of T-lymphocytes against COVID-19 infection (121). However, this may result in boomerang effects given that immune system enhancement can result in a tremendously inflammatory stage–the cytokine storm–to fight against infection caused by SARS-CoV-2. Indeed, the most interesting aspect of ICIs is their ability to improve the “early phase of COVID-19,” thus avoiding possible dangerous immune responses to the viral infection (125).

Encouraging results have also been reported in patients with HIV, HBV, and/or HCV infections treated with ICIs during the COVID-19 pandemic; no adverse effects were noted in these patients in terms of viral infection and non-viral reactivation (126). The TERAVOLT study, an interesting investigation that is still in the preliminary stages, has reported no effects on the survival of patients with cancer treated with chemo-targeted therapies and ICIs and infected with COVID-19 (127). Besides the possible uses of the available medical treatments against cancer, in patients who are ineligible for targeted therapy, targeted ICIs alone or in combination with platinum are considered the gold standard, especially in terms of infection with SARS-CoV-2 (128). Moreover, the use of chemotherapy within one month preceding the diagnosis of COVID-19 is reportedly associated with the occurrence of severe infection and consequent complications (129).

## Conclusions

Considering all the consequences of infection with SARS-CoV-2 on patients with cancer, it is very important to inform and train the COVID-19 frontline workers, including pulmonologists, infectious disease specialists, anesthesiologists, and radiologists, to select the best approaches and treatments, tailored to specific clinical cases. This approach may prove to be extremely beneficial for avoiding further complications related to drug treatment, which may induce immunomodulatory effects favoring cancer and viral infection. However, the data regarding the potential interference between patients with cancer and COVID-19 are not definitive. Consequently, the real dilemma for oncologists is to find a balance between protecting patients using the most effective therapies and reducing the risks of COVID-19 exposure and/or infection. In this direction, the international oncological societies worldwide have set several recommendations to guide clinicians to find a balance in terms of the safety and efficacy of cancer treatment and rescue therapy against viral infections (130). The most interesting approach related to these guidelines is based on the possibility of follow-up or evaluating patients using the telemedicine services to contain the number of patients in hospitals and, consequently, the risk of infection (131, 132). In these 2 years of the pandemic, we have witnessed new approaches and considerations for patients with severe infections as well as those with cancer, who need to be cured in real time despite the serious pandemic situation. This challenging scenario has opened up new avenues for the scientific community to bring innovation and progress further in the field of medicine.

## Author contributions

The idea for the manuscript was conceived by BA and was further developed by VM, AVS. BA, VM, AVS, RT, and SC. EC, FS, and MD reviewed the manuscript All authors contributed to the article and approved the submitted version.

## Acknowledgments

The Authors thank Prozetesis.org for the support.

## Conflict of interest

The authors declare that the research was conducted in the absence of any commercial or financial relationships that could be construed as a potential conflict of interest.

## Publisher’s note

All claims expressed in this article are solely those of the authors and do not necessarily represent those of their affiliated organizations, or those of the publisher, the editors and the reviewers. Any product that may be evaluated in this article, or claim that may be made by its manufacturer, is not guaranteed or endorsed by the publisher.

## Abbreviations

DCCs, dormant cancer cells; NETs, neutrophil extracellular traps; ARF, acute respiratory failure; ARDS, acute respiratory distress syndrome; HFNC, high flow nasal cannula; NIV, noninvasive ventilation; FCR, functional residual capacity; ACE 2, angiotensin-converting enzyme-2; LUAD, lung adenocarcinoma; LUSC, lung squamous carcinoma; TMPRSS2, Transmembrane Serine Protease 2; EMT, epithelial-to-mesenchymal transition; DCs, dendritic cells.
